# Mitigation of Corrosion on Magnesium Alloy by Predesigned Surface Corrosion

**DOI:** 10.1038/srep17399

**Published:** 2015-11-30

**Authors:** Xuming Zhang, Guosong Wu, Xiang Peng, Limin Li, Hongqing Feng, Biao Gao, Kaifu Huo, Paul K. Chu

**Affiliations:** 1 Department of Physics and Materials Science, City University of Hong Kong, Tat Chee Avenue, Kowloon, Hong Kong, China; 2 The State Key Laboratory of Refractories and Metallurgy, Wuhan University of Science and Technology, Wuhan 430081, China

## Abstract

Rapid corrosion of magnesium alloys is undesirable in structural and biomedical applications and a general way to control corrosion is to form a surface barrier layer isolating the bulk materials from the external environment. Herein, based on the insights gained from the anticorrosion behavior of corrosion products, a special way to mitigate aqueous corrosion is described. The concept is based on pre-corrosion by a hydrothermal treatment of Al-enriched Mg alloys in water. A uniform surface composed of an inner compact layer and top Mg-Al layered double hydroxide (LDH) microsheet is produced on a large area using a one-step process and excellent corrosion resistance is achieved in saline solutions. Moreover, inspired by the super-hydrophobic phenomenon in nature such as the lotus leaves effect, the orientation of the top microsheet layer is tailored by adjusting the hydrothermal temperature, time, and pH to produce a water-repellent surface after modification with fluorinated silane. As a result of the trapped air pockets in the microstructure, the super-hydrophobic surface with the Cassie state shows better corrosion resistance in the immersion tests. The results reveal an economical and environmentally friendly means to control and use the pre-corrosion products on magnesium alloys.

Solid-liquid contact is a common phenomenon in our daily lives and the associated scientific principles have also permeated industrial applications. When metals are exposed to an aggressive medium, corrosion often takes place frequently undermining the performance^1^. Magnesium (Mg) is one of the lightest metals and its alloys are attractive to applications requiring light weight and excellent machinability, for instance, the transport, aerospace, and biomedical industry^2^,^3^,^4^,^5^. However, the corrosion resistance of Mg alloys is often not favorable thereby stifling wider utilization. For example, as biodegradable implants, proper control of surface corrosion is crucial to tissue healing after surgery^2^,^6^,^7^,^8^,^9^. In order to control surface corrosion, a viable way is to form a barrier film on the surface to isolate the alloys from the external aggressive environment. For instance, stainless steel has a dense and stable Cr_2_O_3_-rich surface oxide which provides excellent protection^10^. However, the native surface oxide on magnesium formed upon exposure to air consists of mainly MgO and is not stable in aqueous solutions^11^. Alloying is an important way to modify the metallurgical microstructure of magnesium as well as its surface chemistry. For some magnesium alloys, it is believed based on our current knowledge that the corrosion products preformed in the aqueous solution can govern the ensuing corrosion process to a certain extent^12^,^13^. Hence, it is possible to exploit this dense and continuous corrosion product layer with special properties formed *in situ* on the Mg surface to offer protection in a hostile environment. Actually, the difference in the corrosion potentials between different phases or the pristine oxide film and substrate inevitably induces localized galvanic corrosion. Moreover, the formation of the corrosion products is quite complicated and not well understood. For example, when AZ91 Mg alloy is immersed in aqueous solutions, the film on the surface of the α phase consists of three layers: an inner layer rich in Al_2_O_3_, a middle layer consisting mainly of MgO, and an outer layer made of Mg(OH)_2_. The surface film on the *β* phase is different from that on the *α* phase with Mg/Al hydroxide at the solution/film interface and Mg/Al oxide at the alloy/film interface. The Al concentrations in both the inner and outer layers are much larger than that in the film on the *α* phase^11^,^14^,^15^. Consequently, it is challenging to control the formation of corrosion products under laboratory conditions.

Micro- or nano-scale structures are possibly produced on Mg alloys during corrosion in aqueous media^16^,^17^. The microstructured surface is known to play an important role in some super-hydrophobic phenomena in nature such as the lotus leaves effect. Generally, a superhydrophobic surface has a water static contact angle (SCA) greater than 150° and sliding angle (SA) less than 10° and so water droplets roll off the surface easily. The small fraction of the solid/liquid contact area on a superhydrophobic surface gives rise to many air pockets trapped underneath the liquid thereby reducing the actual contact between the bulk materials and external medium^18^,^19^. Obviously, integration of the water-repellent behavior arising from the surface superhydrophobic effect with the sturdy barrier formed by the corrosion products is an effective means to prevent corrosion. However, this concept has seldom be reported and implemented because natural corrosion does not produce a uniform and large-area corrosion product layer with the desirable micro- or nano-scale structures.

Recently, chemical conversion methods have attracted much attention with regard to surface treatment of magnesium alloys. Ishizaki *et al.*^20^ used a steam coating method to prepare Mg-Al layered double hydroxide (LDH) containing magnesium hydroxide on AZ31 magnesium alloy with steam produced from ultrapure water but no special nanostructures were found on the treated surface. They further performed steam coating on AMCa602 alloy with ultrapure water containing Al(NO_3_)_3_·9H_2_O to produce the steam. Although a Mg(OH)_2_/Mg-Al LDH composite film can be formed, the nanostructures are not distributed uniformly on the surface^21^. Zhu *et al.*^22^ selected a hydrothermal method to synthesize the Mg(OH)_2_ film on AZ31 magnesium alloy using a NaOH solution. Similarly, some nanostructures are formed on the surface but they are unevenly distributed. Ou *et al.*^23^ conducted the hydrothermal method using ultrapure water on AZ91 Mg alloy and produced nanosheets to construct a super-hydrophobic surface. Strictly speaking, the formation mechanism described in these reports is corrosion and hence, those processes including hydrothermal treatment may offer a viable means to perform pre-corrosion to fabricate a robust and water repelling surface barrier.

Herein, we describe the *in situ* preparation of a uniform layered surface composed of an inner compact hydroxide layer and tunable top Mg-Al layered double hydroxide (LDH) microsheet on AZ80 Mg alloy under hydrothermal conditions by taking advantage of the intrinsic features of Al-enriched Mg alloys and the formation mechanism is studied. By precisely controlling the hydrothermal temperature, time, and pH, the microsheet surface modified by fluorinated silane can be tailored to the Cassie superhydrophobic state with a vertical shape. Accordingly, a robust barrier and water repelling surface can be produced on Al-enriched Mg alloys by this simple and versatile approach which has commercial potential.

## Results and Discussion

**Figure 1:**
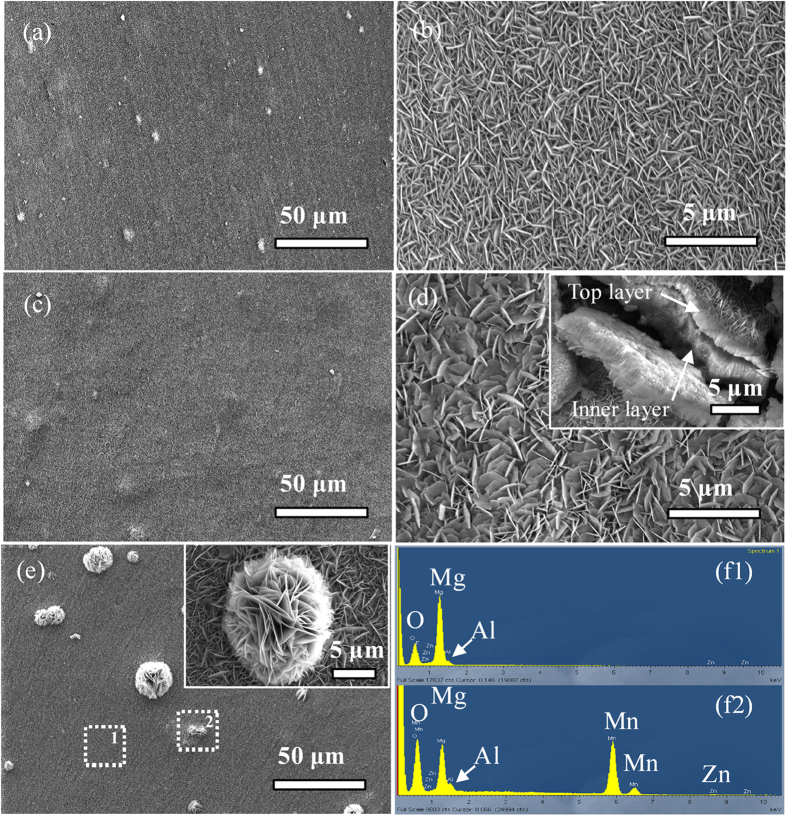
Representative FE-SEM images of the samples treated hydrothermally at 120 °C for (**a**,**b**) 8 h and (**c**,**d**) 12 h at a pH of 12 and (**e**) 120 °C for 12 h in DI Water; (**f**) EDS spectrum acquired from different areas on sample (**e**): (f1) Flat and (f2) Flower-like zones.

Figure 1 shows the low- and high-resolution FE-SEM images of the Mg alloy after the hydrothermal treatment at 120 °C for 8 h (a–b) and 12 h (c–d) at a pH of 12 in aqueous solutions and for 12 h in DI water (pH ≈ 6.5) (e). A uniform film is formed after the hydrothermal treatment at a high pH. Microsheets with a morphology showing a vertical shape about 1 μm in width to a tilted one about 2 μm in width are produced by increasing the reaction time from 8 h to 12 h (Fig. 1b,d). The inset image in Fig. 1d discloses that the hydrothermal coating comprises two different layers composed of an inner compact layer about 4 μm thick and another 1 μm thick microsheet layer on top. If the hydrothermal time is prolonged, the thickness of the layer increases only slightly but the top uniform microsheet layer is partially damaged (Figures S1 and S2). At a high pH (13 and 14), the microsheets become scarce and even disappear resulting in deteriorated corrosion resistance (Figure S2). On the other hand, in DI water, many flower-like structures integrated with many big microsheets are dispersed on the surface in addition to the flat microsheet coating (Fig. 1e). Selected-area EDS shows strong Al and Mn signals from the flower-like structure (Figs 1f1, 2).

**Figure 2:**
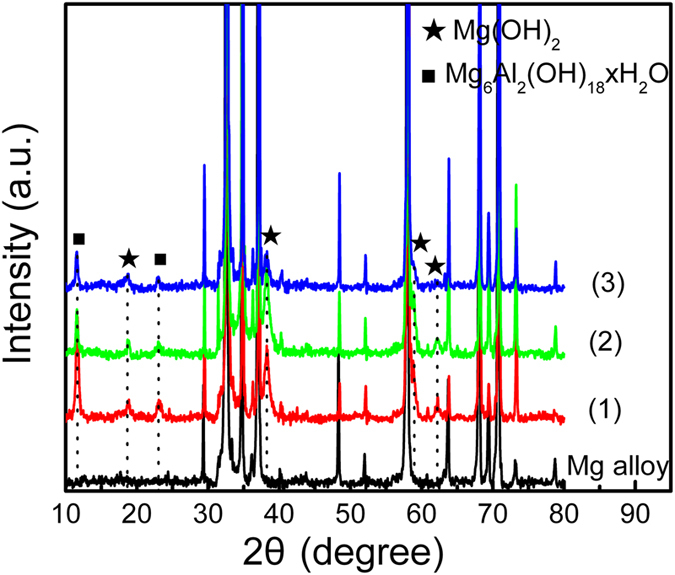
XRD patterns of the hydrothermal Mg alloy: (1) 120 °C for 12 h in DI water, (2) 120 °C for 12 h at a pH of 12, and (3) 120 °C for 8 h at a pH of 12.

The corresponding XRD patterns of the hydrothermal samples are displayed in Fig. 2. With regard to the microsheets prepared on the Mg alloy, the diffraction peaks at 18.7°, 38.5°, 58.9°, and 62.4° can be indexed to the hexagonal Mg(OH)_2_ (JCPDS card: 75–1527) in addition to the Mg alloy substrate^22^. Characteristic peaks at 11.4° and 22.9° attributable to the (003) and (006) diffraction planes of Mg-Al LDH (Mg_6_Al_2_(OH)_18_4H_2_O, JCPDS card: 38–0478)^24^ can also be observed and the intensity goes up with hydrothermal time. It is noted that the XRD peaks of the (003) and (006) planes shift slightly to larger diffraction angles compared to the products inserted by these anions into the interlayer space of Mg-Al LDH such as Mg_1−x_Al_x_(OH)_2_(NO_3_)_x_∙nH_2_O (10.4° and 20.8°)^25^ and Mg_6_Al_2_(OH)_16_CO_3_∙4H_2_O (11.2° and 22.7°)^24^, indicating that the interlayer spacing is influenced by the inserted anions and smaller OH^-^ ions or H_2_O decrease the interlayer spacing resulting in large diffraction angles according to Bragg’s law. The FTIR spectrum in Figure S3 reveals the characteristic vibration of the interlayer water molecules and high basicity of O-H groups in the microsheet layer, further corroborating the formation of the layered Mg-Al LDH structure on the surface.

**Figure 3:**
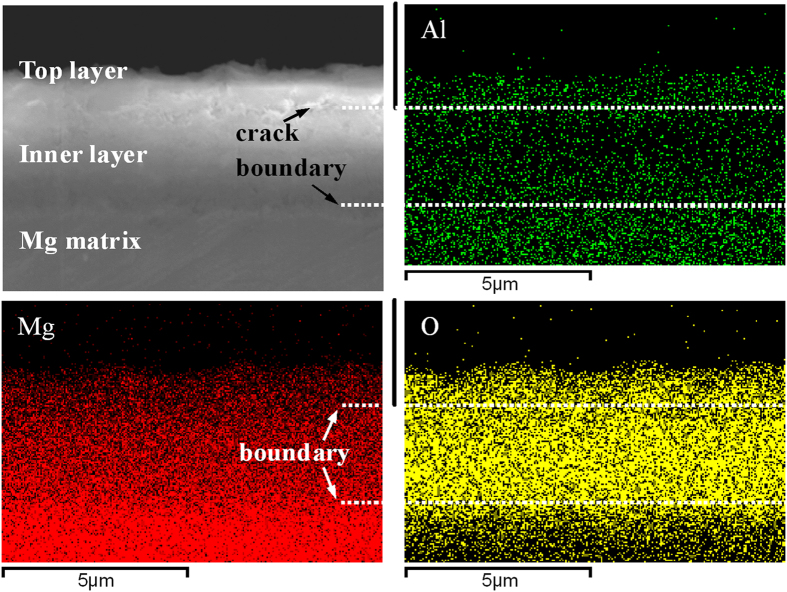
FE-SEM image of the cross-section of the hydrothermal layer coupling with the element mapping of Al, Mg and O (120 °C for 12 h at a pH of 12 in aqueous solutions).

Figure 3 shows the profile of the hierarchical structure and elemental distribution of the cross-section of the hydrothermal layer at 120 °C for 12 h and a pH of 12. A distinct boundary between the Mg matrix and hydrothermal layer and a transverse crack between the top and inner layers are observed from the cross-sectional FE-SEM image. The total thickness of the hydrothermal layer is about 5 μm (according to the oxygen distribution), which is consistent with the hierarchical structure in Figs 1d and S1. The elemental map reveals that Mg and O are uniformly distributed in the hydrothermal layer but aluminum is enriched in the top layer. The inner layer has a small concentration of Al possibly due to Al dissolution, diffusion, and preferential deposition of crystalline Mg-Al LDH at the surface during the long time hydrothermal treatment. It contributes to the growth of the large Mg-Al LDH microsheet. The XPS results acquired from different layers (microsheet and inner condensed layer) indicate that the top microsheet layer is not simply Mg(OH)_2_ and the Mg-OH bond may be associated with Al (Figure S4).

**Figure 4:**
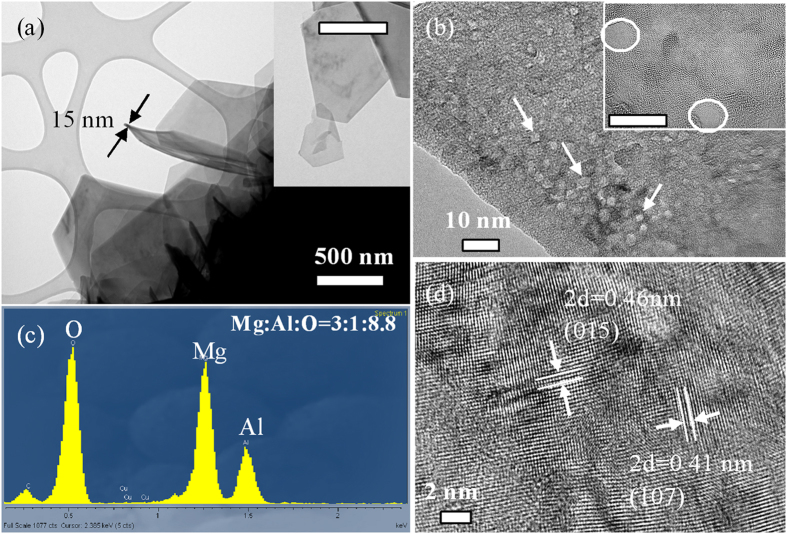
Representative TEM and HR-TEM images and corresponding EDS spectrum of the microsheet. The scale bar in the inset is the same as that in the corresponding TEM image.

Figure 4a depicts the representative TEM images of the microsheets that are composed of many single slices (inset picture) about 15 nm thickness constituting a layered structure. There are many small pits dispersed on the surface of the microsheets as shown by the HR-TEM image in Fig. 4b. The inset atomic-scale HR-TEM image of a single Mg-Al LDH slice discloses defects about 5 nm in diameter. The corresponding EDS spectrum of the microsheets shows strong signals of Mg, Al, and O (carbon signal coming from the substrate) with atomic ratios of 3:1:8.8, suggesting that the Mg-Al LDH has a chemical formula of Mg_6_Al_2_(OH)_18_ (Fig. 4c). The lattice spacings between adjacent planes in the microsheets are approximately 0.23 and 0.205 nm, as shown in Fig. 4d, corresponding to those between the two (015) and (107) crystal planes of Mg_6_Al_2_(OH)_18,_ respectively. The TEM results are consistent with data obtained by XRD, FTIR and XPS confirming the formation of the layered Mg-Al LDH microsheet on the Mg alloy.

The formation mechanism of the uniform hierarchical structure comprising a top Mg-Al LDH microsheet layer and inner compact layer during the one-step hydrothermal treatment is described here. The AZ80 Mg alloy has many non-matrix phases such as the β-phase (Mg_17_Al_12_) and Al_x_Mn_y_ dispersed in the Mg alloy acting as cathodes relative to the Mg matrix (α-phase) causing deleterious effects and affecting the corrosion performance^13^. The primary effect enhances galvanic corrosion of Mg and Al to create supersaturated Mg^2+^ and Al^3+^ around the cathodes which further precipitate to form large flower-like structures which may be potential defects to degrade the corrosion resistance due to the diffusion channels in the non-compact zone. The Mg-Al LDH microsheet layer is conventionally fabricated in acidic solutions, but the formed Mg-Al LDH layer has many cracks compromising the surface uniformity and corrosion resistance^26^,^27^. Here, the film comprises a compact inner layer and uniform Mg-Al LDH microsheet layer as a result of the fast hydrothermal dissolution and precipitation at the high temperature and moderately high pH. Al on the surface of the AZ80 substrate dissolves due to the high OH^−^ concentration, but the high pH also increases the chemical passivity of Mg thus restricting the growth of Mg-Al LDH (Figure S2). The two effects are mediated in the alkalescent solution resulting in precipitation of the uniform Mg-Al LDH microsheet layer on the surface. Even for a short hydrothermal time or low hydrothermal temperature, the precursor of the microsheets can be observed suggesting that precipitation is spontaneous (Figure S5). As the hydrothermal time is increased, the thickness of layer increases slowly because the formed layer inhibits direct contact between the Mg alloy matrix and solution. The chemically active Al in the inner layer dissolves gradually and diffuses to the surface providing the source for the Mg-Al LDH growth (Fig. 3). Costantino *et al.*^28^ have reported that Al ions are co-precipitated with Mg ions to produce a solid substance with a stoichiometry of two Mg^2+^ for each Al^3+^ at pH values of 8.5–9.0. However, a stable structure of layered Mg_6_Al_2_(OH)_18_ with an Mg/Al molar ratio of 3 can be formed preferentially in a high pH environment^24^,^29^, but dissolution of Al from the surface microsheets is also promoted giving rise to many pit defects (Fig. 4). By further increasing the pH to 13 and 14, the Mg-Al LDH microsheets shrink and diminish gradually. It is because of the enhanced chemical passivity of Mg at the high pH thus restricting the growth of Mg-Al LDH. On the other hand, the Al enriched phases such as β-phase (Mg_17_Al_12_) and Al_x_Mn_y_ dissolve to form holes on the surface due to the high chemical activity of Al in the alkaline medium (Figure S2). It should be noted that the pure Mg matrix without Al may not support the growth of microstructures on the Mg substrate because of the small solubility in DI water and strong passivity at high pH (Figure S6).

**Figure 5:**
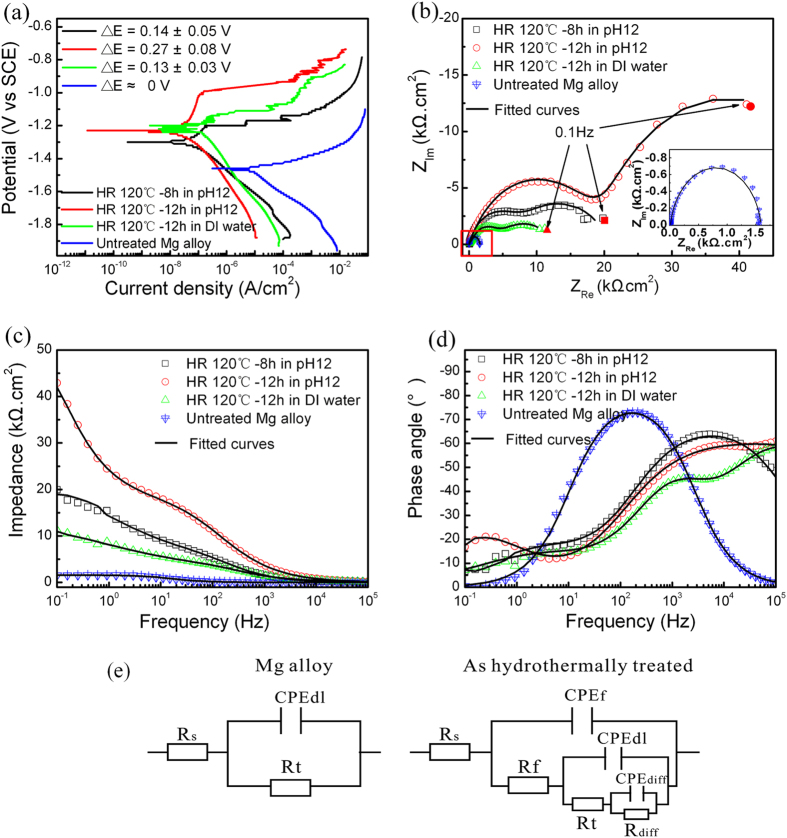
(**a**) Polarization curves of the hydrothermal samples, (**b**) Corresponding Nyquist plots, and (**c,d**) Bode plots. (**e**) Corresponding equivalent circuit model based on the pristine Mg alloy and hydrothermal samples. The measurement is carried out in a 3.5 wt% NaCl aqueous solution.

The corrosion behavior of the hydrothermal coatings (120 °C for 8 h and 12 h at a pH of 12 and 12 h in DI water) is investigated in 3.5 wt% NaCl as shown in Fig. 5. The potentiodynamic polarization curves disclose the improved corrosion resistance by both cathodic hydrogen evolution and anodic dissolution^24^. The hydrogen evolution rate decreases significantly on the cathodic side as well as the enhanced passive region on the anodic side of the hydrothermal samples (Fig. 5a). The polarization measurements show that the corrosion current density (I_corr_) of the hydrothermal samples is (0.55–1.35) × 10^−7^ A.cm^−2^ which is 100–200 times smaller than that of the untreated Mg alloy (1.22 × 10^−5^ A.cm^−2^) thus implying good corrosion resistance. Furthermore, the corrosion current density of the optimal hydrothermal sample (120 °C for 12 h at a pH of 12) demonstrates apparent corrosion resistance improvement compared to the flower-like structure and the sample prepared with a short hydrothermal time in addition to most of the corrosion resistance coatings on Mg alloys such as the Mg-Al LDH layer or Mg-Al LDH and Mg(OH)_2_ composite layer^27^,^30^ and other magnesium oxide layer^23^,^31^,^32^. The excellent corrosion resistance properties can be attributed to the uniform compact and thick inner layer. The potential differences (∆E) between the corrosion potential and transition potential in the anodic region are listed in Fig. 5a indicating a better anti-corrosion behavior for the samples with uniform thick compact inner layers (hydrothermal treatment for 12 h at a pH of 12). Figure 5b–d show the corresponding Nyquist and Bode plots. In EIS, the impedance features fitted by the corresponding equivalent circuits are denoted by R_s_(CPE_f_(R_f_(CPE_dl_(R_t_(CPE_diff_R_diff_)))))^33^,^34^, where R_s_ is the solution resistance between the reference electrode and working electrode. CPE_f_, CPE_dl_ and CPE_diff_ are three time constant phases representing the capacitance of the whole film, electric double layer capacitance, and capacitance pertaining to the diffusion process, and R_f_, R_t_ and R_diff_ denote the resistance of the pores and other defects in the whole film, charge transfer resistance, and diffusion resistance, respectively. Usually, the capacitance CPE_f_, is an important parameter to reflect water absorption in coatings and the resistance R_f_ can be used to measure the porosity and deterioration of the coatings^35^. The experimental data are fitted well by the equivalent model (Fig. 5e) and the calculated values of the individual electrical components with three times replicates are listed in Table 1. Since R_s_ is small and similar in all the tests, it is neglected here. The thick inner compact layer covered by uniform microsheets exhibits larger sums of R_f_, R_t_, and R_diff_ indicative of excellent corrosion resistance in the NaCl solution. The enhanced resistance is also confirmed by the Bode plots including the impendence versus frequency and phase angle versus frequency in Fig. 5c,d.

**Figure 6:**
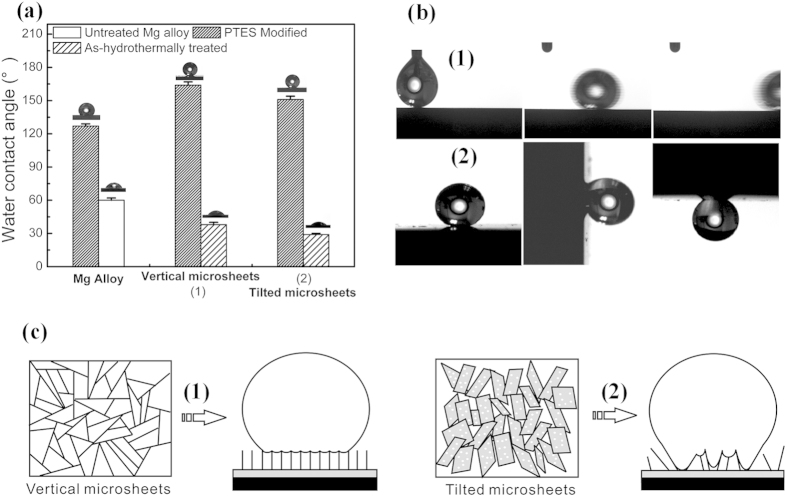
(**a**) Static water contact angles of the different microsheet layers before and after PTES modification: (1) HR 120 °C-8 h at a pH of 12, (2) HR 120 °C-12 h at a pH of 12. (**b**) Photograph of water contact behavior on the vertical and tilted microsheet surfaces. (**c**) Illustration of the patterns of the morphology-induced water contact states on the superhydrophobic surface.

To further improve the corrosion protection, the surface energy is lowered by modification with 1H,1H,2H,2H-perfluorooctyltriethoxysilane (PTES) to obtain a unique superhydrophobic surface^36^. The FTIR spectrum in Figure S3 confirms successful modification of PTES on the surface. The wettability, an important feature to evaluate the relationship between the aqueous solution and substrate, is highly dependent on the surface microstructure. The wetting behavior of the hydrothermal coatings [(1) 120 °C for 8 h, (2) 12 h at a pH of 12] with and without PTES modification is evaluated using an optical water contact angle meter. The hydrothermal surface is hydrophilic showing a small water contact angle of 37 ± 2° on the uniform vertical microsheets layer and 29 ± 1° on the uniform titled microsheet surface. They are smaller than that on the pristine Mg alloy (60 ± 2°). Although the PTES modified pristine Mg alloy exhibits a larger water contact angle (123 ± 2°), surface texture plays an important role in the water repellence effect. Here, superhydrophobic properties for water contact angles over 150° on the hydrothermal samples are observed after PTES modification (Fig. 6a). However, the superhydrophobic surfaces show distinct wettability. When the morphology of the microsheets changes from being vertical to tilted, the contact angles decrease from 164 ± 3° to 153 ± 3° as a result of the increased solid/liquid contact area. The wetting states change from Cassie to Wenzel-like correspondingly (Fig. 6b). It has been shown that the microscopic scale and morphology determine the solid/liquid contact and control the adhesive strength of water droplets on the superhydrophobic surface^36^,^37^. As described by the water contact angle equation proposed by Cassise and Baxter^38^, cos θ′ = f cos θ – (1 − f), where θ′ and θ are the apparent water contact angle on a rough and flat substrate surface respectively. The factor f is the fraction of solid/liquid interface, while (1 − f) is that of the air/liquid interface. Generally, the solid/liquid contact based on the factor f is divided into the area contact, line contact, and point contact, which decide the magnitude of the adhesive force on a superhydrophobic surface^36^. Here, the superhydrophobic surface comprising fine and vertical microsheets (120 °C for 8 h at a pH of 12) shows small sliding angles (<3°), whereas the water droplets on the tilted superhydrophobic microsheet surface (120 °C for 12 h at a pH of 12 or in DI water) oppose the gravitational force when the surface is tilted vertically (90°) or even turned upside down (180°). The distinct water adhesion can be attributed to the different Van der Waals attraction on the surface micro/nanostructures. The vertical microsheet surface provides the line contact with a small contact area resulting in the low adhesive force but on the other hand, the tilted microsheets offer a large contact area which dramatically increases the water adhesive force^36^, as illustrated in Fig. 6c. Meanwhile, there are many pits with several nanometers in diameter on the microsheets might be formed by Al dissolution at a high pH and they are believed to be an important factor of the large adhesive force because the Van der Waals interaction can be strengthened due to the strong dipole moment produced at the edge of the crystal defect^39^,^40^.

**Figure 7:**
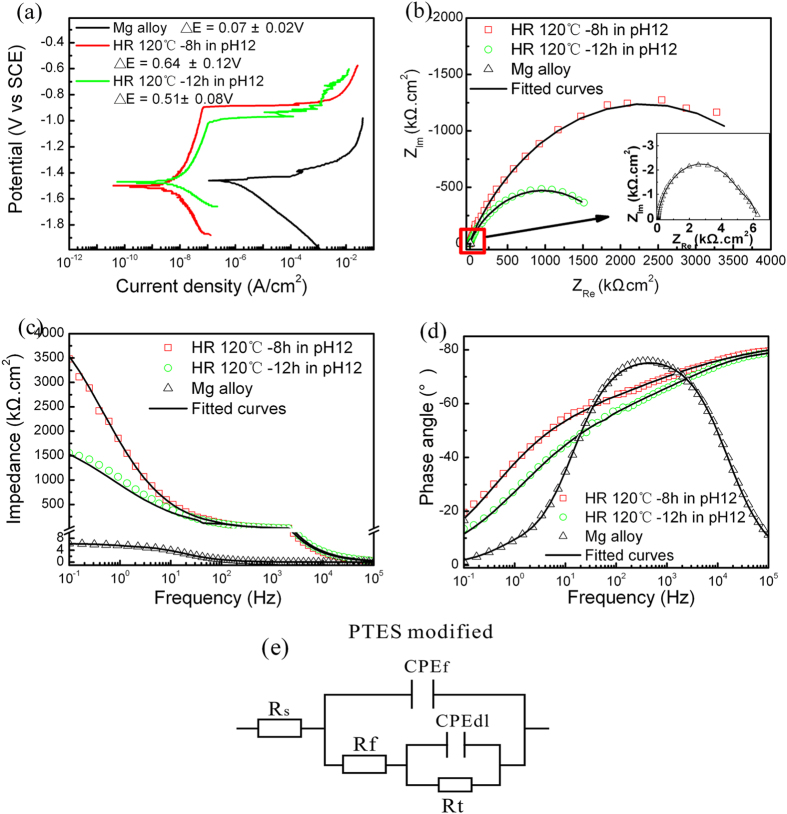
(**a**) Polarization curves of the Wenzel-like (HR 120 °C-12 h at a pH of 12) and Cassie state (HR 120 °C-8 h at a pH of 12) superhydrophobic samples, (**b**) Corresponding Nyquist plots, and (**c,d**) Bode plots. (**e**) Corresponding equivalent circuit model based on the PTES modified samples. The measurement is carried out in a 3.5 wt% NaCl aqueous solution.

Polarization is adopted to determine the instantaneous corrosion rate. As shown in Fig. 7, a smaller corrosion current density and bigger corrosion potential difference (∆E) correspond to a lower corrosion rate and better corrosion resistance thereby providing indication of better corrosion resistance. Notably, I_corr_ [(3.35–9.23) × 10^−9^ A.cm^−2^] obtained from the both superhydrophobic states samples is 10–20 times smaller than that of hydrothermal samples [I_corr_ = (0.55–1.35) × 10^−7^ A.cm^−2^] and ∆E is about three times larger. The corrosion current density of the sample in Cassie-Baxter model is about three times smaller than that in Wenzel-like state in NaCl aqueous solution, and far lower than that of most irregularly rough superhydrophobic surfaces on Mg alloy^18^,^31^,^32^,^41^,^42^,^43^,^44^, the excellent corrosion resistance performance is due to the regular roughness and orientation of microsheets which extremely decrease the chance of contact between the substrate and solution^23^.

EIS of the two superhydrophobic states surface demonstrates that the capacitive loops of the Cassie state superhydrophobic layer is greatly enlarged compared to the Wenzel-like state one. According to the physical structure of the superhydrophobic surface, an equivalent circuit model of R_s_(CPE_f_(R_f_(CPE_dl_R_t_))) with two time constants is utilized to fit the data (Fig. 7e)^45^.The constant phase elements CPE_f_ and CPE_dl_ represent the capacitance of the contact layer and electric double layer, respectively, corresponding to the pores and other defects in the contact layer (R_f_) and charge transfer resistance (R_t_). Owing to smaller penetration of water into the superhydrophobic surface, the equivalent electrical components associated with the diffusion process are negligible. The fitted EIS data are listed in Table 1. R_f_ and R_t_ are about one and three orders of magnitude larger than those of the hydrothermal samples, respectively, and the Cassie state superhydrophobic surface has the largest R_t_ or sum of R_f_ and R_t_ thus showing the best corrosion resistance. The good results can be attributed to that when a water droplet is placed on the superhydrophobic surface with a smaller fraction of solid/liquid contact area f, the trapped air pockets underneath the liquid occupying the apparent surface area lead to stronger surface water-repellence thereby reducing the actual contact between the bulk materials and external medium. Conversely, the water droplets on the Wenzel-like state surface can easily extrude the air pockets and probably contact the inner compact layer.

The durability of the Wenzel-like and Cassie state superhydrophobic surfaces in a corrosive environment is crucial to practical applications. After immersion in 3.5 wt% NaCl for 7 days, the Cassie state superhydrophobic surface continues to offer effective corrosion protection compared to the Wenzel-like state and no white corrosion products appear on the surface, as shown in Figure S7. Furthermore, the FE-SEM image discloses that the Cassie state superhydrophobic surface still retains the vertical microsheets on the surface despite the deformation of partial Mg-Al LDH microsheets (Figure S8).

## Conclusion

A novel concept involving a pre-corrosion treatment is described and it offers an economical and convenient means to produce a double-barrier structure on Mg alloys to protect against aqueous corrosion. The hierarchical coating composed of an inner compact hydroxide layer and top uniform Mg-Al LDH microsheets on the Mg alloy is prepared by a one-step hydrothermal process in water. The hydrothermal Mg alloy possesses good corrosion resistance in the neutral saline solution. By adjusting the hydrothermal time, temperature, and pH, the orientation of the top microsheets can be tailored from being tilted to vertical and consequently, the superhydrophobic state is changed from the Wenzel-like one to Cassie one after modification with fluorinated silanes. The Cassie-state superhydrophobic surface enhances the long-term corrosion resistance because the air pockets trapped in the surface microstructures act as the ideal hydrophobic medium.

## Experimental Section

### Materials

The AZ80 Mg alloy samples with dimensions of 10 × 10 × 5 mm^3^ contained 8 wt% Al, 0.5 wt% Zn, and 0.2 wt% Mn. The samples were ground by 2,000 grit SiC paper, ultrasonically cleaned in ethanol, and dried in nitrogen ambient. The PTES [1H,1H,2H,2H-perfluorooctyltriethoxysilane, CF_3_(CF_2_)_5_CH_2_CH_2_Si(OCH_2_CH_3_)_3_], analytical grade NaCl, and NaOH were obtained from sigma-Aldrich Co. and used without further treatment.

### Hydrothermal fabrication of hierarchical barrier layers

The cleaned Mg alloy samples were laid flat in 10 ml of aqueous solutions with different pH values in a 25 ml Teflon-lined autoclave. The pH of the solution was adjusted by NaOH (0.01 M, 0.1 M, and 1 M). The autoclave was sealed and heated in an oven to different temperature for different time durations at a rate of 10 °C min^−1^. After the hydrothermal reaction (HR), the autoclave was cooled down in air automatically, and the specimens were removed from the vessel, washed with DI water and ethanol several times, and dried in air at room temperature. After drying for one day, the hydrothermally treated samples were immersed in methanol containing hydrolyzed 1 wt% PTES for 2 h and heated to 140 °C for 1 h in air to remove the residual PTES.

### Characterization

The samples were characterized by X-ray diffraction with Cu K_α_ radiation (λ = 1.5418 Å) (XRD, Philips X’ Pert Pro), field-emission scanning electron microscopy in conjunction with energy-dispersive X-ray spectroscopy (FE-SEM, FEI Nova 400 Nano and EDS, Oxford INCA 200), transmission electron microscopy (TEM) (Philips CM20), and high-resolution TEM (HR-TEM) (JEM-2010F). Chemical bonding information was collected by Fourier transform infrared spectroscopy (FTIR) (Perkin Elmer 1600). The water contact angles (WCA) and sliding angles were measured with a water droplet volume of 4 μL on an optical apparatus (Ramé-Hart Model 250 Standard Goniometer).

### Electrochemical measurements

The electrochemical experiments were performed on a Zahner Zennium electrochemical workstation using the conventional three-electrode technique in 3.5 wt% NaCl at room temperature. A saturated calomel electrode (SCE) served as the reference electrode, a platinum foil was the counter electrode, and the hydrothermally treated samples were exposed as the working electrode. After immersion for 1 h, electrochemical impedance spectroscopy (EIS) was conducted from 100 mHz to 100 kHz with a 10 mV sinusoidal perturbing signal at the open-circuit potential and polarization tests were conducted subsequently at a scanning rate of 1 mV s^−1^. To evaluate the long-term corrosion resistance, the pristine Mg alloy and hydrothermal samples before and after acquiring superhydrophobicity were immersed in 3.5 wt% NaCl solutions at room temperature for 7 days.

## Additional Information

**How to cite this article**: Zhang, X. *et al.* Mitigation of Corrosion on Magnesium Alloy by Predesigned Surface Corrosion. *Sci. Rep.* **5**, 17399; doi: 10.1038/srep17399 (2015).

## Supplementary Material

Supplementary Information

## Figures and Tables

**Table 1:** Fitted data from the electrochemical impedance spectra (EIS) of untreated, hydrothermal, and PTES modified Mg alloy.

Samples	R_s_ (Ωcm^2^)	CPE_f_ (Ω^−2^ cm^2^ s^−n^)	R_f_ (Ωcm^2^)	CPE_dl_ (Ω^−2^ cm^2^ s^−n^)	R_t_ (Ωcm^2^)	CPE_diff_ (Ω^−2^ cm^2^ s^−n^)	R_diff_ (Ωcm^2^)
Untreated Mg alloy	10.29 ± 0.07			(1.42 ± 0.31)×10^−5^	(2.63 ± 0.91)×10^3^		
HR120 °C-12 h DI Water	9.78 ± 0.28	(2.78 ± 0.52)×10^−7^	(8.05 ± 0.32)×10^2^	(1.13 ± 0.43)×10^−6^	(3.91 ± 0.75)×10^3^	(4.51 ± 0.87)×10^−5^	(1.12 ± 0.34)×10^4^
HR120 °C-12 h pH12	10.12 ± 0.89	(4.43 ± 1.89)×10^−7^	(2.81 ± 1.42)×10^4^	(3.36 ± 2.57)×10^−6^	(4.79 ± 1.82)×10^3^	(5.67 ± 4.25)×10^−5^	(5.98 ± 3.67)×10^4^
HR120 °C-8 h pH12	11.21 ± 0.56	(7.31 ± 0.64)×10^−7^	(4.72 ± 3.8)×10^3^	(1.81 ± 1.31)×10^−5^	(5.10 ± 3.27)×10^3^	(3.98 ± 1.74)×10^−5^	(1.95 ± 0.79)×10^4^
PTES- Mg alloy	10.41 ± 0.91	(1.46 ± 0.58)×10^−6^	(1.67 ± 0.05)×10^3^	(7.04 ± 0.93)×10^−5^	(4.78 ± 0.11)×10^3^		
PTES-HR120 °C-12 h pH12	13.54 ± 3.91	(1.32 ± 1.26)×10^−8^	(1.97 ± 0.27)×10^4^	(1.87 ± 0.31)×10^−7^	(1.37 ± 0.54)×10^6^		
PTES-HR120 °C-8 h pH12	15.33 ± 5.96	(1.48 ± 1.07)×10^−8^	(1.61 ± 0.69)×10^4^	(1.43 ± 0.15)×10^−7^	(7.68 ± 3.4)×10^6^		
