# Supplementary material for: Mitigation of Corrosion on Magnesium Alloy by Predesigned Surface Corrosion
Source: Sci Rep. 2015 Nov 30;5:17399. doi: 10.1038/srep17399 (PMC4663789; doi:10.1038/srep17399)
Supplement: Supplementary Information [file srep17399-s1.doc]

**Supporting Information on line:**

**Mitigation of Corrosion on Magnesium Alloy by Predesigned Surface Corrosion**

Xuming Zhang,^1^ Guosong Wu,^1*^ Xiang Peng,^1^ Limin Li,^1^ Hongqing Feng,^1^ Biao Gao,^2^ Kaifu Huo,^1,2^ Paul K. Chu^1*^

^1^ Department of Physics and Materials Science, City University of Hong Kong, Tat Chee Avenue, Kowloon, Hong Kong, China

^2^ The State Key Laboratory of Refractories and Metallurgy, Wuhan University of Science and Technology, Wuhan 430081, China

* Correspondence and requests for materials should be addressed to:

[paul.chu@cityu.edu.hk](mailto:paul.chu@cityu.edu.hk) (PK Chu) or [guosonwu](mailto:guosongwu@cityu.edu.hk)[@cityu.edu.hk](mailto:hochen@cityu.edu.hk) (GS Wu)

*
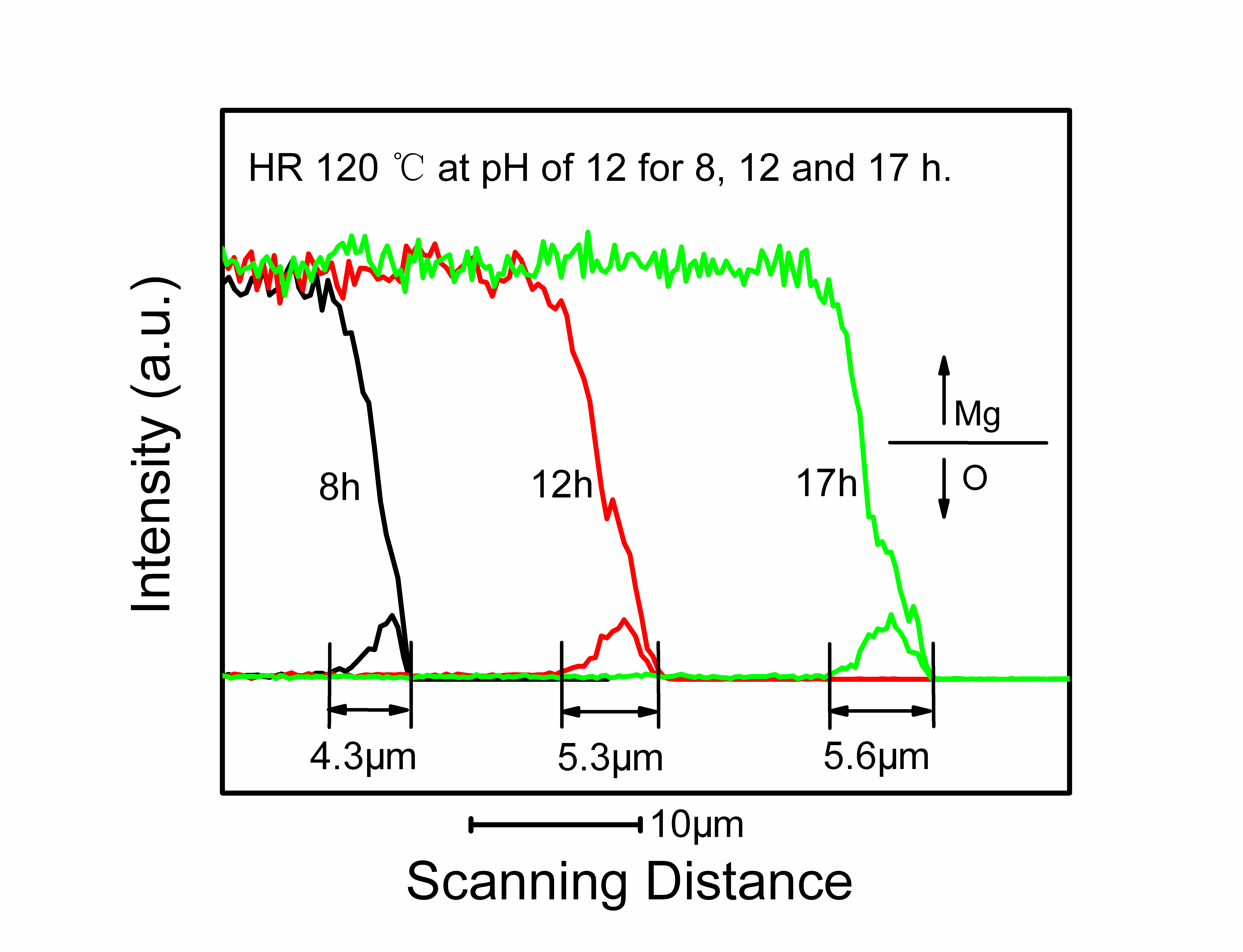
*

**Figure S1.** EDS line scans conducted on the cross-sectioned hydrothermal layers prepared at 120 °C and pH of 12 for 8, 12 and 17 h.

**Figure S1** depicts the EDS line scans of the cross sections of the hydrothermal layers. The thickness increases slowly with hydrothermal time because the formed hydrothermal layer inhibits direct contact between the Mg matrix and solution resulting in a small formation rate of hydroxide on the interface. The thickness is affected by water penetration as a thicker hydrothermal layer allows less water penetration leading to slower layer growth.


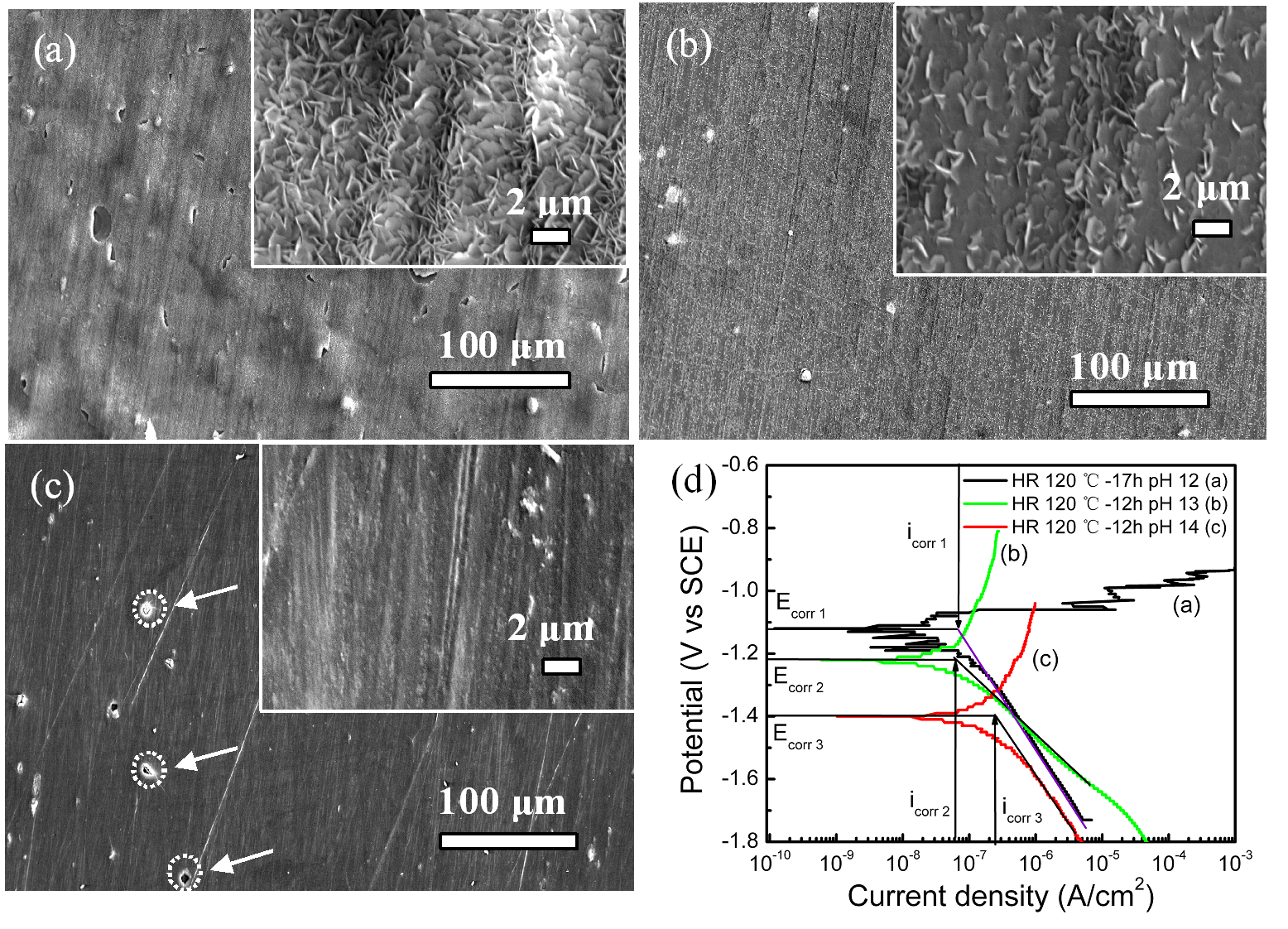


**Figure S2.** SEM images of the hydrothermal samples: (a) 120 °C for 17 h at a pH of 12 , (b) 120 °C for 12 h at a pH of 13, (c) 120 °C for 12 h in a pH of 14, and (d) Corresponding polarization plots.

**Figure S2** illustrates the morphological evolution of the samples under different hydrothermal conditions. The top uniform microsheet layer is partly damaged when the hydrothermal time is 17 h possibly due to dissolution of Al in the top Mg-Al hydrotalcite layer at a relative high pH. At a pH of 13, the microsheets become scarce and disappear at a pH of 14. It is because of the enhanced passivity of Mg at high pH values restricting the growth of Mg-Al hydrotalcite. The Al enriched phases such as β-phase (Mg_17_Al_12_) and Al_x_Mn_y_ dissolve due to the high chemical activity of Al in an alkaline solution and holes remain on the surface causing corrosion resistance deterioration.


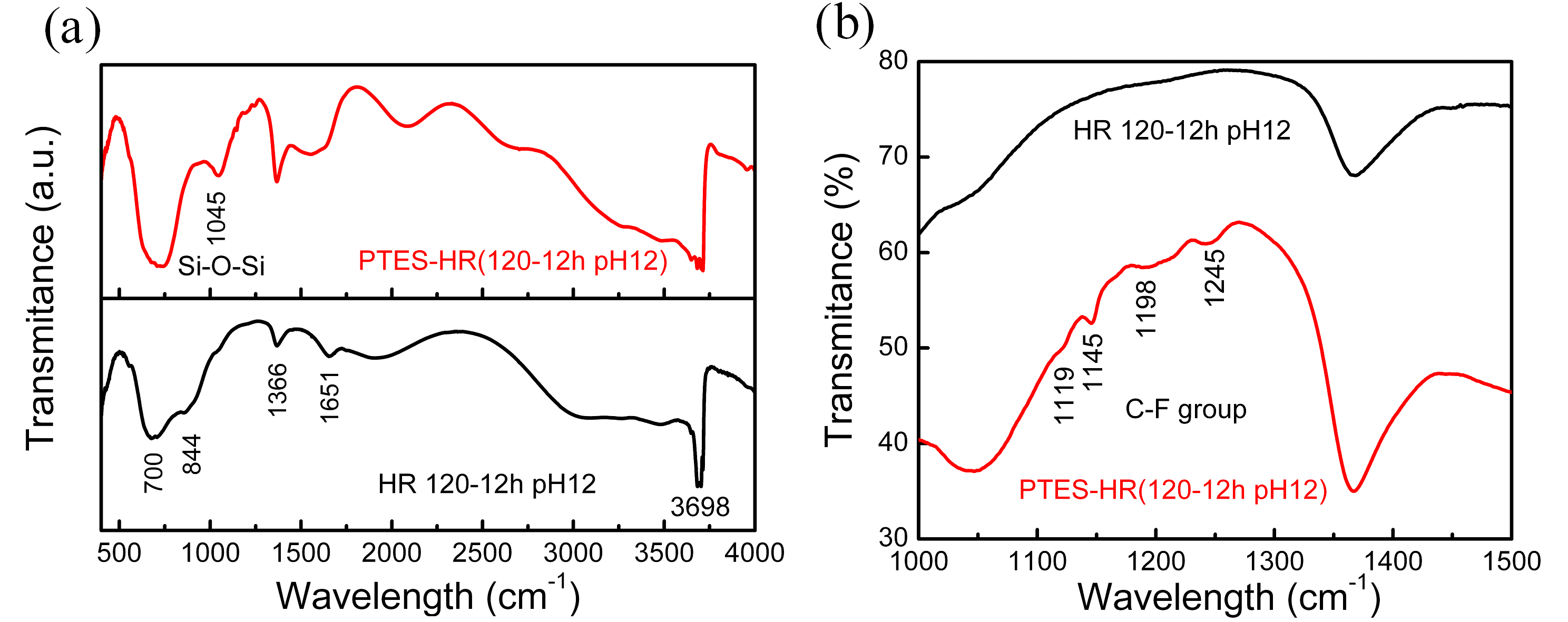


**Figure S3.** FTIR spectra of the microsheet layer before (black line) and after (red line) PTES modification: (a) Full spectra from 400 to 4000 cm^-1^ and (b) Fine spectra from 1000 to 1500 cm^-1^.

The FTIR spectra of the hydrothermal layer before and after PTES modification acquired at a resolution of 2 cm^-1^ are displayed in **Figure S3**. Before modification, there is a single sharp band at 3,698 cm^-1^ attributable to the high basicity of O-H groups in the surface microsheets.^1^ The weak broad band around 1650 cm^-1^ can be assigned to deformation vibration of interlayer water molecules in hydrotalcite.^2^ The band around 1366 cm^-1^ corresponds to asymmetrical stretching of CO_3_^2-^ in the interlayer due to carbon dioxide dissolved in water.^2^ Generally, the lattice vibration of metal-oxygen bonds can be observed at around 650 cm^-1^ but a broad and intense bond around 700 cm^-1^ is observed. The crystal defects in the microsheets may increase the dipole moment of the chemical bonds and induce blue-shifts.^3^ After PTES modification, several bonds emerge. An intense adsorption band in the range between 1020 and 1150 cm^-1^ suggests the formation of Si–O–Si bond on the surface.^4^ The presence of C-F groups is detected in the range of 1300-1100 cm^-1^ and the adsorption bands of C-F_2_ are at 1245, 1198, 1145, and 1119 cm^-1^, respectively.^5^


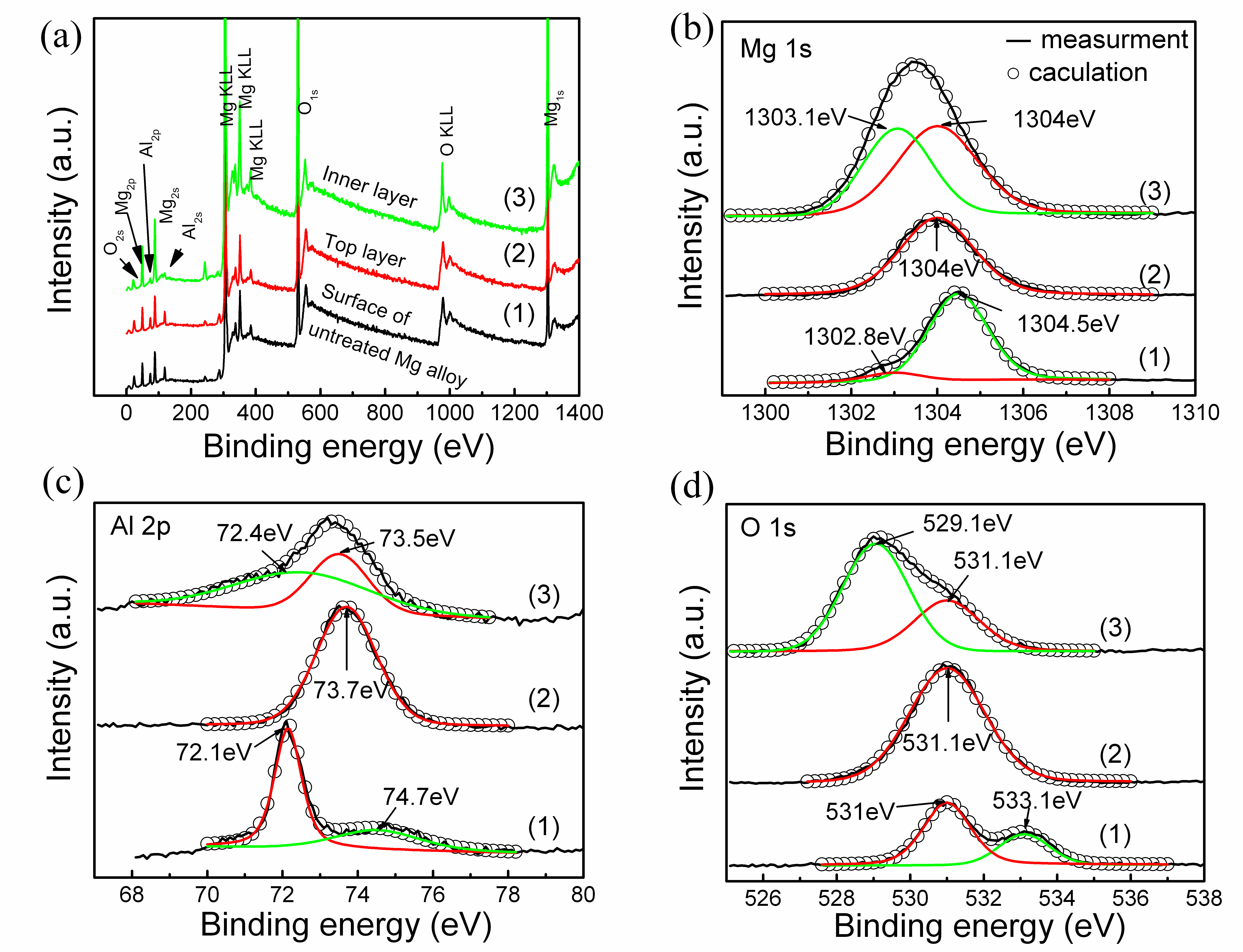


**Figure S4.** Full and fine XPS spectra: (1) Untreated Mg alloy surface, (2) Top microsheet layer, and (3) Inner compact layer.

The XPS spectra in **Figure S4** are used to determine the in-depth chemical composition of the layers. Al, Mg, and O (oxygen) are detected but not Mn and Zn due to the low concentrations of the latter. The high-resolution XPS spectra of Al 2p, Mg 1s, and O 1s acquired from the untreated Mg alloy (1) reveal the hydroxide and oxide states, which are 1302.8 and 1304.5 eV for Mg in Mg(OH)_2_ and MgO, 72.1 and 74.7 eV for Al in Al(OH)_3_ and Al_2_O_3_, and 531 and 533.1 eV for O in metal-OH and adsorbed water, respectively.^6-8^ The spectra of Mg 1s, Al 2p, and O 1s obtained from the microsheet layer (2) can be fitted with binding energies of 1304 eV, 73.7 eV, and 531.1 eV corresponding to the binding energies of Mg^2+^, Al^3+^ in the octahedral structure as well as lattice oxygen or metal-OH.^9,10^ Hence, it is not simply Mg(OH)_2_ and the Mg-OH bond may be associated with Al. With regard to the inner compact layer (3), slight shifts from Mg(OH)_2_ (1302.8 eV) and Al(OH)_3_ (72.1 eV) to 1303.1 eV and 72.4 eV can be observed and the strong peak of O 1s at a smaller binding energy of 529.1 eV can be ascribed to the presence of O^2-^,^11^ suggesting abundant Mg and Al sub-hydroxide in the layer due to the incomplete hydrothermal reaction at large depths. XPS reveals that the hydrothermal layer is composed of top Mg-Al compounds and an inner layer containing abundant sub-hydroxide and residual Mg-Al compounds.


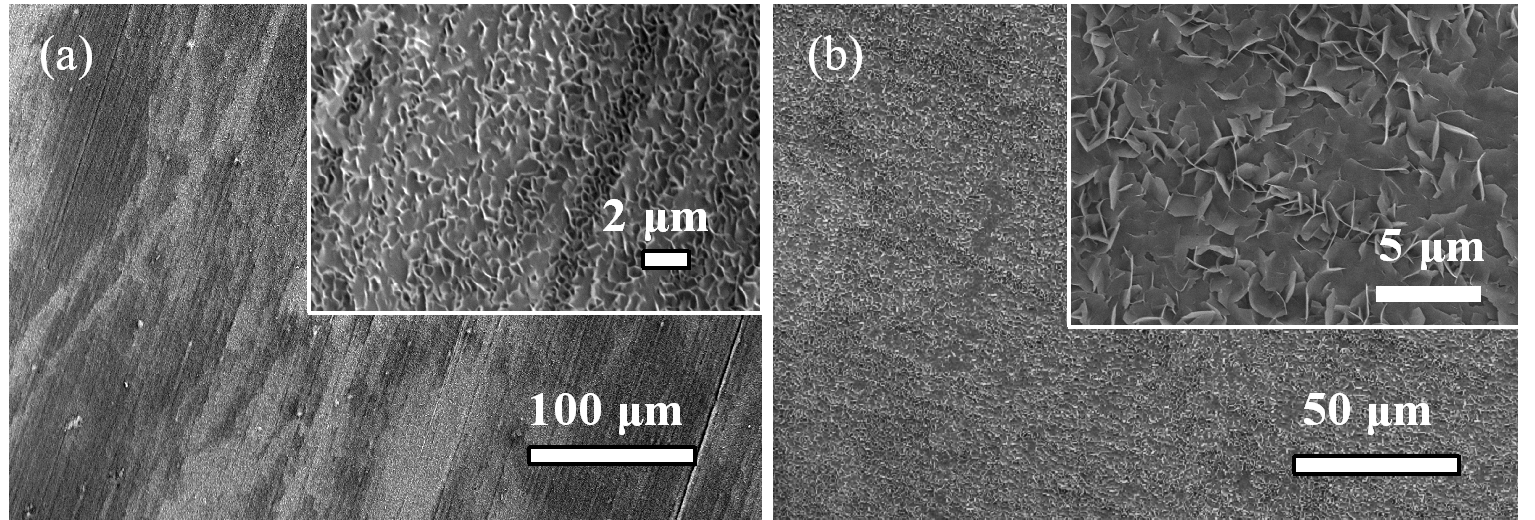


**Figure S5.** SEM images of the hydrothermal samples: (a) 120 °C for 4 h and (b) 100 °C for 12 h at pH of 12.

**Figure S5** shows the surface morphology after a short hydrothermal time or under a low temperature. For the short hydrothermal time (a), the wrinkled structure can be observed and as time elapses, the microsheet structure grows and becomes denser. At a relative low hydrothermal temperature (b), the microsheets are formed and stacked on the surface. Both of them indicate spontaneous growth of microsheets during the hydrothermal treatment.


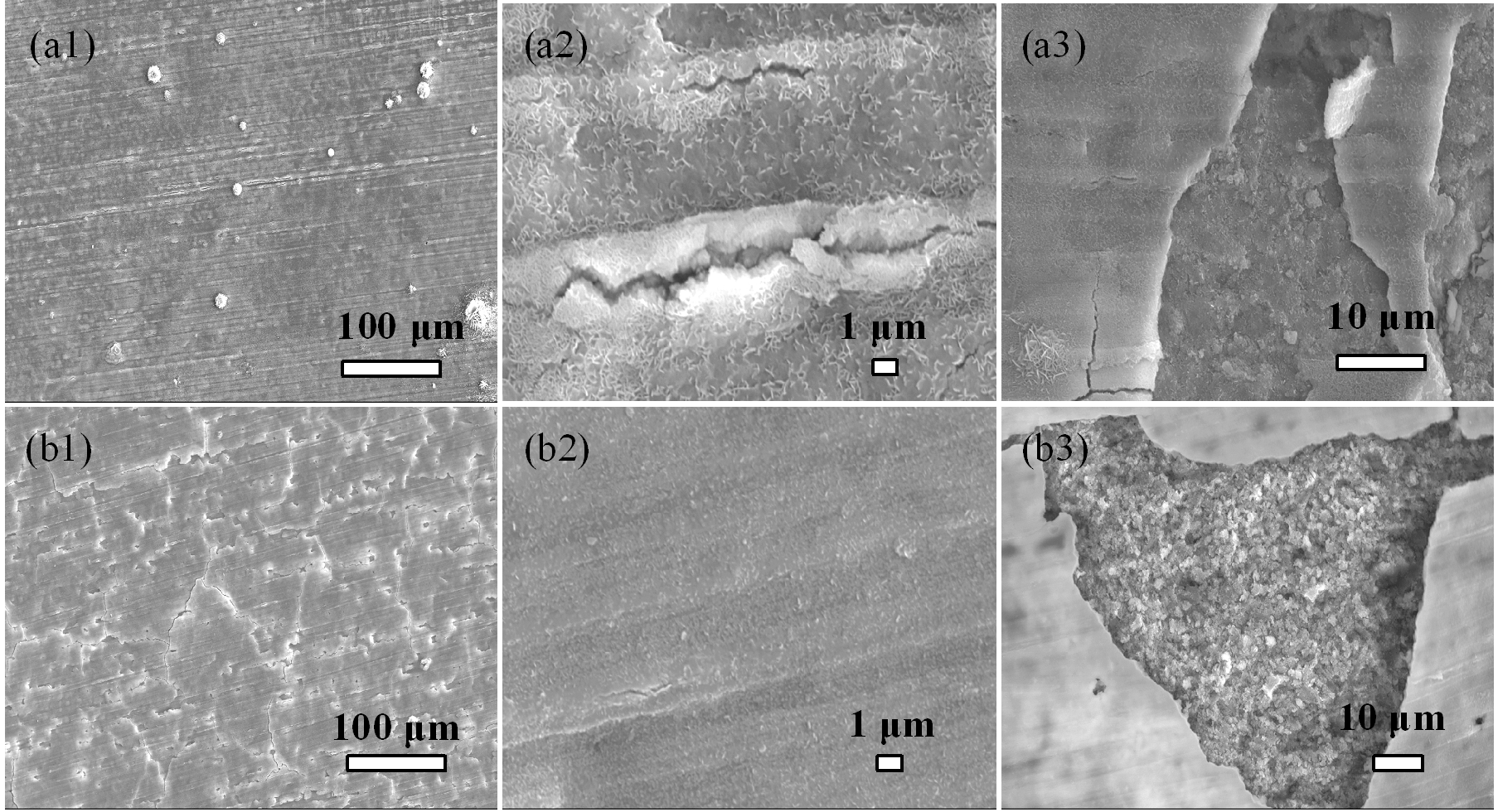


**Figure S6.** SEM images of pure Mg substrate after hydrothermal treatment under different conditions: (a) 120°C for 12 h in DI water and (b) 120°C for 12 h in pH 12.

**Figure S6** depicts the surface features on the pure Mg samples (without Al) after the hydrothermal treatment in water at a pH of 12. Owing to the chemical passivity of Mg in alkaline solutions, the Mg^2+^ concentration is smaller than that in water resulting in slightly different morphologies. The smaller Mg^2+^ concentration induces a smooth and condensed surface. Few flower-like structures can be observed after the hydrothermal treatment in water and small wrinkled microstructures and visible cracks are observed from the flat area. It indirectly indicates that the AZ80 magnesium alloy can produce high concentrations of Mg^2+^ and Al^3+^ for the growth of the microstructures since non-matrix phases such as β-phase (Mg_17_Al_12_) and Al_x_Mn_y_ can enhance corrosion of the matrix *via* the galvanic effect.


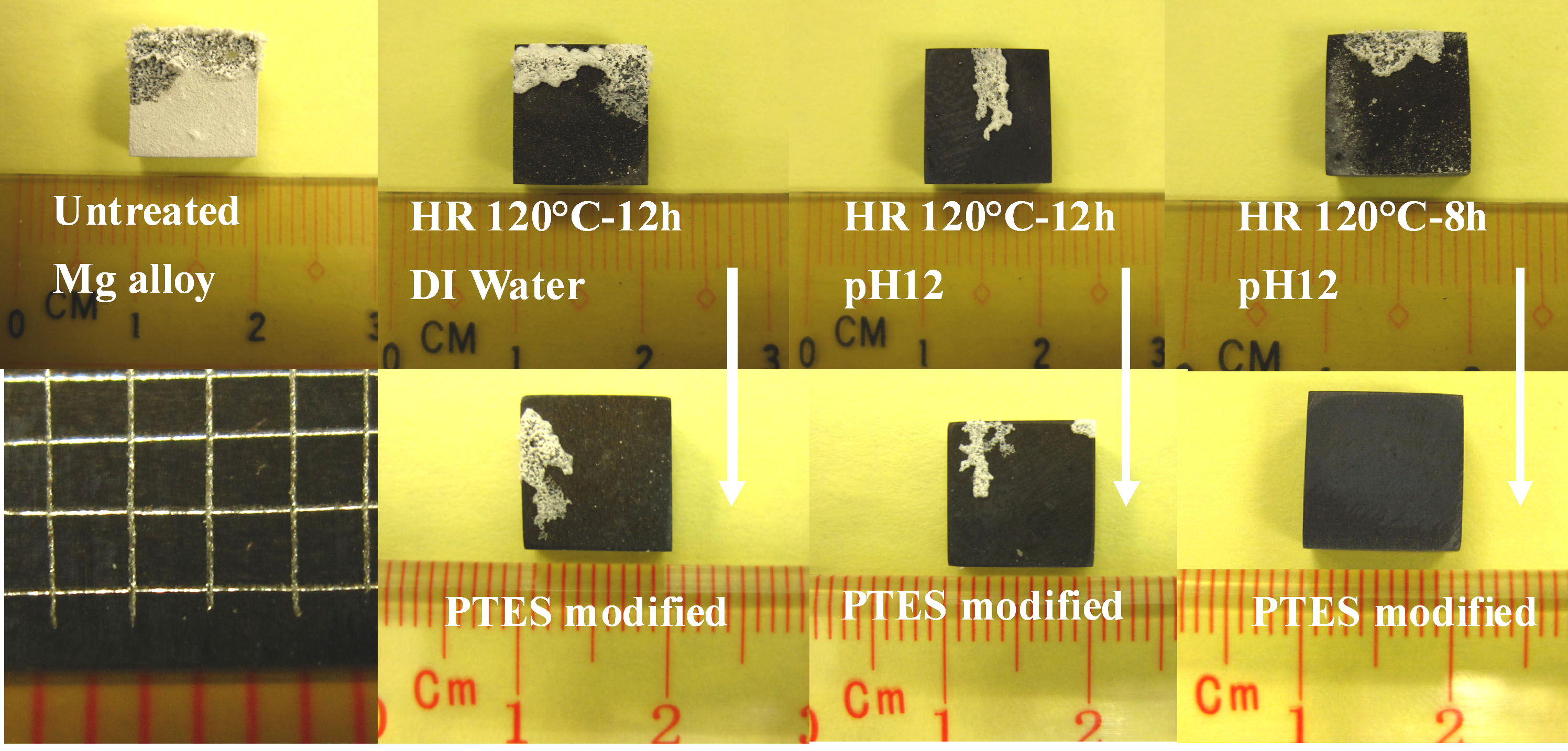


**Figure S7.** Digital images of the hydrothermal samples before and after PETS modification after immersion in a 3.5 wt% NaCl solution for 7 days.

To assess adhesion of the hydrothermally formed film on the Mg alloy, the cross-cut tape test is performed according to ASTM D 3359-02 method B, as shown in **Figure S7**. The film shows good adhesion on the Mg alloy surface without delamination or detachment at the edges and within the square lattice. After immersion in 3.5 wt% NaCl for 7 days, the morphologies of the samples are shown by the digital photographs. The hydrothermal samples show some localized corrosion at the edge of the coating and the naked AZ80 substrate suffers severe damage. After PTES modification, the Cassie state superhydrophobic surface (PTES modified HR 120°C-8h at a pH of 12) continues to offer effective corrosion protection compared to the Wenzel-like state and no white corrosion products can be observed from the surface.


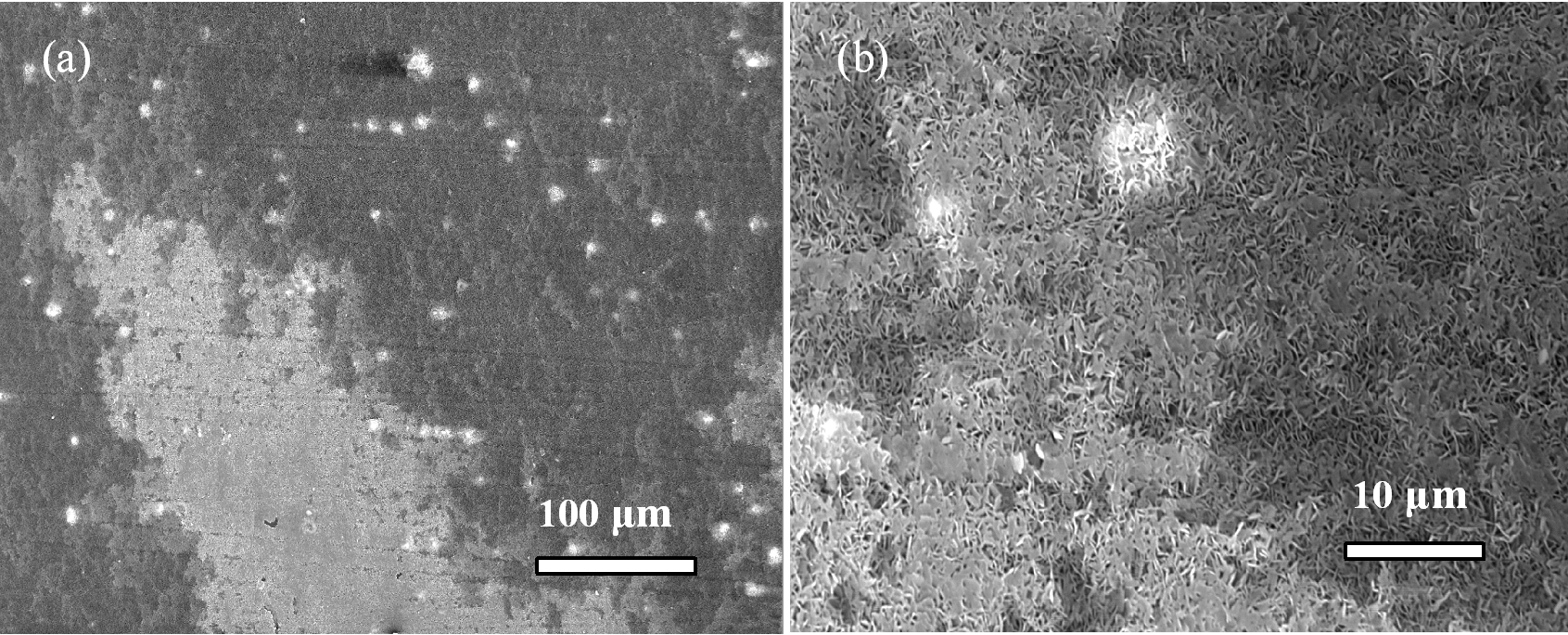


**Figure S8.** SEM images of the sample with the Cassie state superhydrophobic surface (PTES modified HR 120 °C-8h at a pH of 12) after immersion in the 3.5 wt% NaCl solution for 7 days: (a) Low-resolution image and (b) High-resolution image.

The FE-SEM images in **Figure S8** clearly show that after immersion, the Cassie state superhydrophobic surface retains the effective air pockets despite partial deformation of the Mg-Al LDH microsheets.

**References**

1. T. Ishizaki, S.-P. Cho and N. Saito, *CrystEngComm*, 2009, **11**, 2338-2343.

2. J. Chen, Y. W. Song, D. Y. Shan and E. H. Han, *Corros. Sci.*, 2013, **74**, 130-138.

3. F. W. DelRio, M. P. de Boer, J. A. Knapp, E. David Reedy, P. J. Clews and M. L. Dunn, *Nat. Mater*, 2005, **4**, 629-634.

4. V. V. Tomina, G. R. Yurchenko, A. K. Matkovsky, Y. L. Zub, A. Kosak and A. Lobnik, *J. Fluor. Chem.*, 2011, **132**, 1146-1151.

5. S. Pazokifard, S. M. Mirabedini, M. Esfandeh and S. Farrokhpay, *Adv. Powder Technol.*, 2012, **23**, 428-436.

6. G. S. Wu, Y. Zhao, X. M. Zhang, J. M. Ibrahim and P. K. Chu, *Corros. Sci.*, 2013, **68**, 279-285.

7. M. Liu, S. Zanna, H. Ardelean, I. Frateur, P. Schmutz, G. L. Song, A. Atrens and P. Marcus, *Corros. Sci.*, 2009, **51**, 1115-1127.

8. A. F. Lucrédio, G. T. Filho and E. M. Assaf, *Appl. Surf. Sci.*, 2009, **255**, 5851-5856.

9. M. M. Rao, B. R. Reddy, M. Jayalakshmi, V. S. Jaya and B. Sridhar, *Mater. Res. Bull.*, 2005, **40**, 347-359.

10. J. Chen, Y. W. Song, D. Y. Shan and E. H. Han, *Corros. Sci.*, 2011, **53**, 3281-3288.

11. J. Chen, Y. W. Song, D. Y. Shan and E. H. Han, *Corros. Sci.*, 2012, **63**, 148-158.
